# Differential induction of apoptosis and autophagy by pyrrolizidine alkaloid clivorine in human hepatoma Huh-7.5 cells and its toxic implication

**DOI:** 10.1371/journal.pone.0179379

**Published:** 2017-06-26

**Authors:** Wenju Liu, Xu Li, Bo Zhou, Shoucai Fang, Wenzhe Ho, Hui Chen, Hao Liang, Li Ye, Jun Tang

**Affiliations:** 1 Key Laboratory of Combinatorial Biosynthesis and Drug Discovery (Wuhan University), Ministry of Education, and Wuhan University School of Pharmaceutical Sciences, Wuhan, P. R. China; 2 Guangxi Key Laboratory of AIDS Prevention and Treatment & Guangxi Collaborative Innovation Centre for Biomedicine, School of Public Health, Guangxi Medical University, Nanning City, Guangxi, P. R. China; 3 Department of Pathology and Laboratory Medicine, Temple University School of Medicine, Philadelphia, PA, United States of America; 4 Geriatrics Digestion Department of Internal Medicine, The First Affiliated Hospital of Guangxi Medical University, Nanning City, Guangxi, P. R. China; Duke University School of Medicine, UNITED STATES

## Abstract

Growing evidence suggests that the pyrrolizidine alkaloids (PAs)-induced hepatotoxicity is mediated by multiple cell death/defence modalities. However, the detailed mechanisms are still lacking. In this study, the hepatotoxic effects of four PAs including three retronecine-type ones (senecionine, seneciphylline and monocrotaline) and one otonecine-type (clivorine) on the proliferation of Huh-7.5 cells and the possible mechanisms were investigated. The results showed that all the PAs could inhibit cell proliferation and induce apoptosis in a concentration-dependent manner. Among them clivorine was the most significant one. In addition to its effect on apoptosis, clivorine treatment could promote autophagy in Huh-7.5 cells, as evidenced by the accumulation of autophagosomes, the enhancement of LC3B expression at the concentrations close to its IC_0_ value, and the increased conversion of LC3B-I to LC3B-II in the presence of lysosomal inhibitor (chloroquine) and decreased formation of green fluorescent protein (GFP)-LC3 positive puncta in the presence of autophagic sequestration inhibitor (3-methyladenine). Among the other tested PAs, senecionine and seneciphylline also activated autophagy at the same concentrations used for clivorine but monocrotaline did not. Furthermore, our study demonstrated that suppression or enhancement of autophagy resulted in the remarkable enhancement or suppression of senecionine, seneciphylline and clivorine-induced apoptosis at the concentration close to the IC_10_ for clivorine, respectively, indicating a protective role of autophagy against the PA-induced apoptosis at the low level of exposure. Collectively, our data suggest that PAs in different structures may exert different toxic disturbances on the liver cells. Apoptosis may be one of the most common models of the PA-induced cytotoxicity, while autophagy may be a structure-dependent defence model in the early stage of PA intoxication. Differential induction of apoptosis and autophagy probably depending on the concentration is essential for the cytotoxic potency of clivorine.

## Introduction

**Fig 1:**
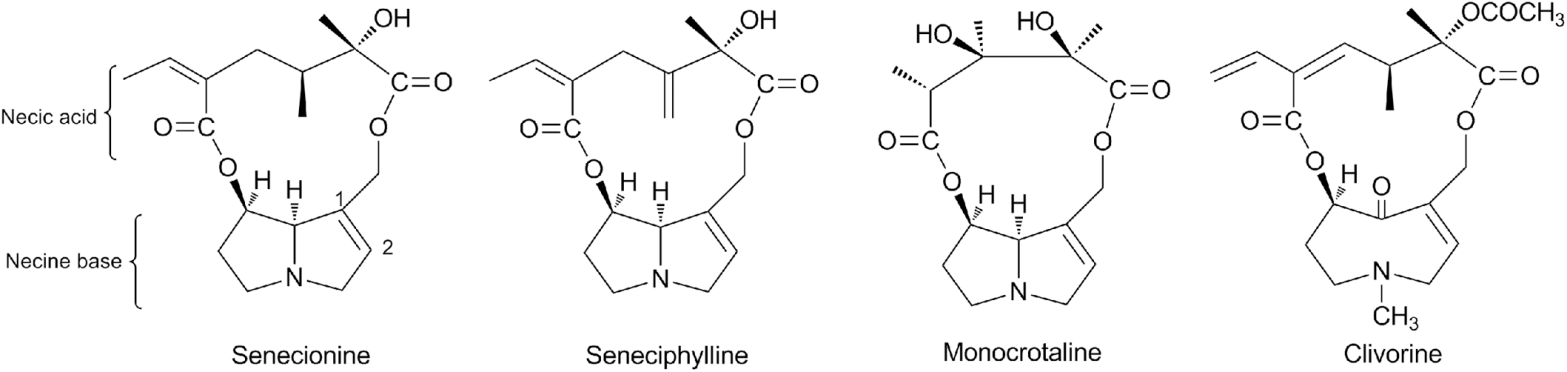
The chemical structures of senecionine, seneciphylline, monocrotaline and clivorine.

Pyrrolizidine alkaloids (PAs) are widely distributed in about 3% of the world’s flowering plants mainly from three families: Boraginaceae, Asteraceae, and Fabaceae [1, 2]. Broadly, more than 660 PAs and PA *N*-oxides have been identified so far, which are mainly divided into three types: retronecine-type, otonecine-type and platyphylline-type [3, 4]. The former two types (Fig 1) are believed to have hepatotoxicity [4–6], genotoxicity [5], neurotoxicity [7, 8], pneumotoxicity [5, 9] and embryotoxicity [10]. The apparent insults in humans after exposure of these PAs have been manifested as hepatic sinusoidal obstructive syndrome (HSOS) accompanied by hepatic megalocytosis, ascites, jaundice, and chronic cirrhosis [5, 6]. To data, there have been numerous HSOS case reports of human intoxication through PA-containing herbs or foodstuffs consumptions in many countries, but there is yet no definite treatment for it [6, 11–13].

Senecionine, seneciphylline and monocrotaline are three representatives for retronecine-type PAs. The former twos are commonly found in genera such as *Senecio* and *Gynura* of the family Asteraceae, while monocrotaline is mainly present in *Crotalaria* of the family Fabaceae [3, 11]. For example, senecionine and seneciphylline are two major PA components in the roots of *Gynura japonica* (Thunb.) Juel., which is used for the treatment of physical injuries and traumatic haemorrhages in Chinese herbal medicines (CHM) [11, 12]; monocrotaline is a predominant PA in the seeds of *Crotalaria assamica* Benth., which has effects of dispelling wind-damp syndrome, stopping bleeding and diminishing swelling [9]. As a representative otonecine-type PA, clivorine is abundant in quite a few *Ligularia* species from Asteraceae, whose roots and rhizomes are usually used to relieve cough and remove excessive phlegm in CHM [13–15]. Currently, intake or misuse of PA-containing herbs for medical purposes is still common in China, creating a growing safety concern with the potential exposure to PAs [3, 11–16].

In view of the mechanism of PA-induced hepatotoxicity, PAs may suffer metabolic activation by cytochromes P450 (CYP450s) to produce the highly reactive pyrrolic metabolites and then cause hepatotoxicity [17–19]. However, how the PAs, either retronecine or otonecine-type, induce hepatotoxicity at the cellular and molecular levels is not well known. In the past twenty years, a few modes of cell death, *e*.*g*., necrosis (oncosis) and apoptosis have been found to mediate the PA-induced cytotoxicity [13, 20–22]. Several retronecine-type PAs including senecionine, monocrotaline, isoline, retrorsine and riddelliine and the otonecine-type PA clivorine had been reported to cause apoptosis *in vitro* and/or *in vivo* [20–23]. For instance, Ji *et al*. [20, 24] found that clivorine could remarkably inhibit cell proliferation and induce mitochondria-mediated apoptosis in human liver L-02 cells. This effort involved the pathway by degradation of Bcl-xL protein, mitochondrial release of cytochrome *c*, and activation of caspases-3/-9. The further study indicated that senecionine also involved the same apoptotic signalling pathway as clivorine [20]. Monocrotaline could induce both oncosis and apoptosis of hepatic parenchymal cells of rats *in vivo* which but occurred differently in distribution [22]. The oncotic lesions primarily occurred in the centrilobular regions with abundant CYP450s, while the caspase inhibition could prevent the development of both oncosis and apoptosis with little effects on the bioactivation of monocrotaline. A hypoxia-regulated cell-death factor, BNIP3, was found to be up-regulated and implicated in switching the mode of cell death from apoptosis to oncosis after monocrotaline exposure. The study on retrorsine showed that its cytotoxic mode on Huh-7 cells may be dose-dependent with apoptosis at low doses and necrosis at high doses [21]. A recent study also found that the PC12 cells after clivorine exposure involved the apoptotic death at the concentrations higher than 50 μM while suppressed neuronal differentiation via TrkA/Akt signalling pathway at lower doses than it [7]. All the evidence suggests that the modes of PA-induced cell toxicities were complex and diverse with involvements of many cellular factors and/or events, which may depend on chemical structure, concentration, treatment time and cell types or even cellular compartment.

Autophagy (hereafter referring to macroautophagy) is the naturally destructive mechanism that disassembles, through a regulated process, unnecessary or dysfunctional cellular components [25]. During this process, targeted cytoplasmic constituents are isolated from the rest of the cell within a double-membraned vesicle known as an autophagosome. The autophagosome then fuses with a lysosome and the contents are degraded and recycled [26]. In the context of disease, autophagy has been seen as an adaptive response to stress, which promotes survival, whereas in some other cases it appears to stimulate cell morbidity and death, or sometimes called autophagic cell death [27]. Many studies have showed that progress of many diseases, *e*.*g*., tumour, diabetes and drug-induced liver injuries (DILI) are closely related to the autophagic process in cells [28–30]. In the case of DILI, autophagy was found to protect the liver cells by removal of the damaged mitochondria, which regulation may be a new approach for the treatment of liver diseases [31, 32], but if too much damaged mitochondria overload autophagy or elicit “autophagic stress”, the DILI may be enhanced [33, 34]. In China, the PAs-containing herbal medicines have been an important cause of DILI owing to numerous HSOS cases [6, 16]. Even though some modes of cell death and signal pathways, *e*.*g*. oncotic necrosis and mitochondrial-mediated apoptosis have been reported [20–24], there is still limited knowledge of the liver cell-intrinsic response modes and machineries for the PA-induced toxicity.

In our preliminary study, clivorine could modulate the mRNA expression of the autophagy-associated gene *LC3* (corresponding to microtubule-associated protein 1 light chain 3) in Huh-7 cells. The evidence implies that autophagy may have an impact on the toxicity of PAs. In the present study, we continued to study the toxic effects of PAs on the human hepatoma Huh-7.5 cells with three retronecine-type PAs (senecionine, seneciphylline, monocrotaline) and one otonecine-type PA clivorine at different concentrations. Their effects on cell proliferation and underlying mechanism especially involving autophagy were investigated. Our findings demonstrate that all PAs have cytotoxic potency, among them, the most distinct one is clivorine. The same apoptotic pathway may be responsible for their toxicities, while autophagy may play a protective role in the early stage of toxic insults by PAs especially clivorine.

## Materials and methods

### Chemicals and reagents

Senecionine and seneciphylline were isolated from *Gynura japonica* (Thunb.) Juel., clivorine was from *Ligularia hodgsonii* Hook., and monocrotaline was from *Crotalaria assamica* Benth. as previously described [3, 9, 14]. All PAs' structures were confirmed using MS and NMR spectroscopy and their purities further determined to be more than 98% by HPLC analyses. Dulbecco's modified eagle medium (DMEM) were purchased from Corning Co., Ltd. (Corning, NY, USA); fetal bovine serum (FBS) were purchased from Gibco/ Thermo Fisher Scientific China (Shanghai, China). Chloroquine (CQ), rapamycin (Rapa) and 3-methyladenine (3-MA) were purchased from Sigma-Aldrich (Shanghai, China). MTT cell proliferation and cytotoxicity assay kits were purchased from Boster Co. Ltd. (Wuhan, China). Annexin V-kFluor488/PI double staining Apoptosis Detection Kit were purchased from KeyGen BioTech Co. Ltd. (Shanghai, China). Both reverse transcriptase kit and qPCR kit were purchased from TAKARA biotechnology Co. Ltd. (Dalian, China). The primers were purchased from Sangon Biotech Co., Ltd. (Shanghai, China). All other reagents were purchased from Sigma-Aldrich (St. Louis, MO) or Sinopharm Chemical Reagent Co. Ltd. (Shanghai, China), unless otherwise indicated. Prior to the experiments, the 0.1 M stock solutions of senecionine, seneciphylline, monocrotaline and clivorine were prepared by dissolving in 10% DMSO aqueous solution.

### Cell line

Human cancer liver Huh-7.5 cells were kindly provided by Professor Wenzhe Ho at Temple University School of Medicine, USA. The cells were maintained in Dulbecco’s modified Eagle’s medium (DMEM) supplemented with 10% fetal bovine serum (FBS), 100 U/mL penicillin, and 100 μg/mL streptomycin at 37°C with 5% CO_2_.

### Cell viability assay

Cells in log-phase were seeded in 96 well plates (10, 000 cells/well) overnight and then treated with senecionine, seneciphylline, monocrotaline and clivorine for 48 h. The cells without PA treatments (with only solvent) were used as the negative controls. Eleven concentrations were set for each PA, including 0 μM, 3.125 μM, 6.25 μM, 12.5 μM, 25 μM, 50 μM, 100 μM, 200 μM, 400 μM, 800 μM and 1000 μM; each repeated for 6 wells. Cell viability was assayed using the MTT assay kit according to the standard procedure provided by the supplier. Briefly, 22 μL of MTT staining fluid was added in each well, and then incubated for 4 h. Afterwards, 100 μL of the formazan crystals were dissolved by adding 2-propanol in each well. The absorption of the formazan solution was measured by a Multiskan GO spectrophotometer (Thermo Scientific China, Shanghai, China) at a wavelength of 570 nm [35]. The cell viability was calculated as the following formula.

$$\mathrm{The}\;\mathrm{cell}\;\mathrm{viability}\;(\%)=\frac{\mathrm{The}\;\mathrm{OD}\;\mathrm{value}\;\mathrm{of}\;\mathrm{PA - treated}\;\mathrm{group}}{\mathrm{The}\;\mathrm{OD}\;\mathrm{value}\;\mathrm{of}\;\mathrm{control}\;\mathrm{group}}\times 100$$

Based on the values of cell viability, the values of IC_50_ (the concentration that inhibited cell viability to 50% of the control), IC_20_ (the concentration that inhibited cell viability to 20% of the control), IC_10_ (the concentration that inhibited cell viability to 10% of the control) and IC_0_ (the maximal concentration without inhibition on the cell viability) were also determined using nonlinear regression analysis in GraphPad Prism 4 (GraphPad Software, Inc., LaJolla, USA).

### Flow cytometry

For flow cytometry analysis, four concentrations were selected for each PA, including 6.25 μM, 25 μM, 100 μM, and 400 μM. Most of the concentrations were lower than the calculated IC_50_ value for each PA (except 400 μM for clivorine) to avoid the non-apoptotic cell death. After treatment, the cells were collected, washed with PBS and digested by 0.25% trypsin solution (without EDTA), and then further incubated at room temperature for 2 minutes. After that, the cells were re-collected and suspended with binding buffer. The suspension was incubated with Annexin V-kFluor488 and stained with PI (Kaiji Biology Corp., China) at room temperature for 20 minutes and then analyzed on flow cytometer (Cyto Flexz, Beckman, USA). The annexin V-positive/PI-negative cells indicated apoptotic cells at the early period, and the annexin V-positive/PI-positive cells showed those at the later period [36]. The ratio of apoptosis or apoptosis index (AI) was calculated using the following equation:

$$\mathrm{AI}\;(\%)=\frac{\mathrm{Apoptosis}\;\mathrm{cell}\;\mathrm{numbers}}{\mathrm{Apoptosis}\;\mathrm{cell}\;\mathrm{numbers}+\mathrm{the}\;\mathrm{normal}\;\mathrm{cells}}\times 100$$

### Real-time PCR analysis

SYBR Green quantitative real-time PCR (RT-PCR) was used to investigate the mRNA expression levels of the autophagy-associated genes including *LC3*, *ATG3*, *ATG5*, and *ATG7*. For this analysis, 24 well plates were used by seeding 50, 000 cells (500 μL of cell suspension) per well, which were then treated with or without 56 μL of each PA for 24 h. Three concentrations for each alkaloid, respectively, including 3.125 μM, 6.25 μM and 12.5 μM were used for the experiments. All concentrations were in or lower than the range of the calculated IC_0_ and IC_10_ values for each PA to avoid the undesirable cytotoxicity. Total RNA was extracted with TRIzol, and the first strand cDNA was synthesized with TAKARA RR036A reverse transcriptase kit following the manufacturer’s instructions. Real-time PCR was performed with the Bio-Rad CFX96 thermal cycler (Bio-Rad Laboratories, Hercules, CA, USA) according to the manufacturer’s protocol. Primers for all autophagic genes and the internal reference GAPDH were designed by Primer express 2.0, which sequences were as follows:

- *GAPDH* (internal reference) sense, 5'-*GGT GGT CTC CTC TGA CTT CAA CA*-3' and antisense 5'-*GTT GCT GTA GCC AAA TTC GTT GT*-3';
- *LC3* sense, 5'-*AGC AGC ATC CAA CCA AAA TC*-3' and antisense 5'-*CTG TGT CCG TTC ACC AAC AG*-3';
- *ATG3* sense, 5'-*CCA ACA TGG CAA TGG GCT AC*-3' and antisense 5'-*ACC GCC AGC ATC AGT TTT GG*-3';
- *ATG5* sense, 5'-*TGG GAT TGC AAA ATG ACA GA*-3' and antisense 5'-*TTT CCC CAT CTT CAG GAT CA*-3';
- *ATG7* sense, 5'-*CAC TGT GAG TCG TCC AGG AC*-3' and antisense 5'-*CGC TCA TGT CCC AGA TCT CA*-3'.

Each cycle threshold (Ct) was measured and normalized to the average Ct value of GAPDH (n = 6). The results were calculated by 2^-△△ct^ method and given as the relative transcriptional levels of target genes.

### Western-blot analysis

In this experiment, three concentrations (3.125 μM, 6.25 μM, and 12.5 μM) were also chosen for each PA, respectively, according to RT-PCR results. Total protein concentrations were determined by NanoDrop2000 spectrophotometer (Thermo Scientific, USA), for which each sample was normalized to the equal total protein concentration. Proteins were separated by SDS-PAGE and probed with appropriate primary and secondary antibodies as previously described by Li *et al*. [37]. The following antibodies including anti-human antibodies against LC3B (1:3000, Cell Signalling), and internal reference β-actin (1:5000, Millipore) were used. The protein levels including LC3B-I, LC3B-II and β-actin were quantified by Quantity One (Chemical light emitting gel imaging system).

### Transmission electron microscope analysis

Electron microscopy was utilized to detect the autophagy response in Huh-7.5 cells treated with or without senecionine, seneciphylline, monocrotaline and clivorine (each 6.25 μM). The cells were digested with trypsin and centrifuged, and then immediately fixed with 2.5% glutaraldehyde/0.1 M sodium cacodylate. The cells were then post-fixed with 1% osmium tetroxide, followed by dehydration with an increasing concentration gradient of ethanol and propylene oxide. The cells were then embedded with the epoxy resin Epon812 and cut to ultrathin sections (50–60 nm) using an ultramicrotome (LKB-I, Bromma, Sweden). Images were examined using a H7650 electron microscope (Hitachi Ltd., Tokyo, Japan) at 80 kV after the samples were stained with 3% uranyl acetate and lead citrate.

### Fluorescence microscopic analysis

Transient GFP-LC3-expressing Huh-7.5 cells were generated using lentivirus-mediated GFP-LC3 over-expression. A lentiviral vector containing GFP-LC3 fusion gene and lentivirus were purchased from Genechem Company (Shanghai, China). Infection of Huh-7.5 cells with lentivirus was carried out at a multiplicity of infection (MOI) of 10.0. Autophagy was assessed using GFP-LC3 redistribution in cells that was detected by an inverted fluorescence microscope (Nikon Ti-s, Japan). The number of GFP-LC3 puncta per cell was determined in three independent experiments (30 cells were counted per experiment).

### Autophagy inhibitor and activator treatments

To address the PA-induced autophagy flux in Huh-7.5 cells, the autophagy inhibitors were used. Briefly, CQ (5 μM) and 3-MA (5 mM) were added to the cells naive and expressing GFP-LC3 from lentiviruses, respectively, 2 h before senecionine, seneciphylline and clivorine treatments (each 6.25 μM). The PA-treated cells were then subjected to the measurement of autophagy-related protein levels by Western blotting or of green fluorescence signal by fluorescence microscope as described above. As a further effort to address the relationship between apoptosis and autophagy, both the autophagy inhibitor 3-MA and autophagy activator Rapa were used. Huh-7.5 cells were pre-treated with the 3-MA (5 mM), or Rapa (100 nM) for 2 h, and then incubated in the presence of senecionine, seneciphylline, clivorine and monocrotaline (each 12.5 μM) for 24 h. Finally, the cells were harvested and treated for the flow cytometry analysis.

### Statistical analysis

Values are expressed as mean ± SD of at least three independent experiments. Statistical analysis was performed using SPSS 17.0 software (SPSS Inc., Chicago, IL) or GraphPad Prism 4 (GraphPad Software, Inc., LaJolla, USA), using one-way analysis of variance followed by two-tailed *t*-test for evaluation, *P* < 0.05 was considered to be significantly different.

## Results

### Cytotoxicity of senecionine, seneciphylline, monocrotaline and clivorine in Huh-7.5 cells

**Fig 2:**
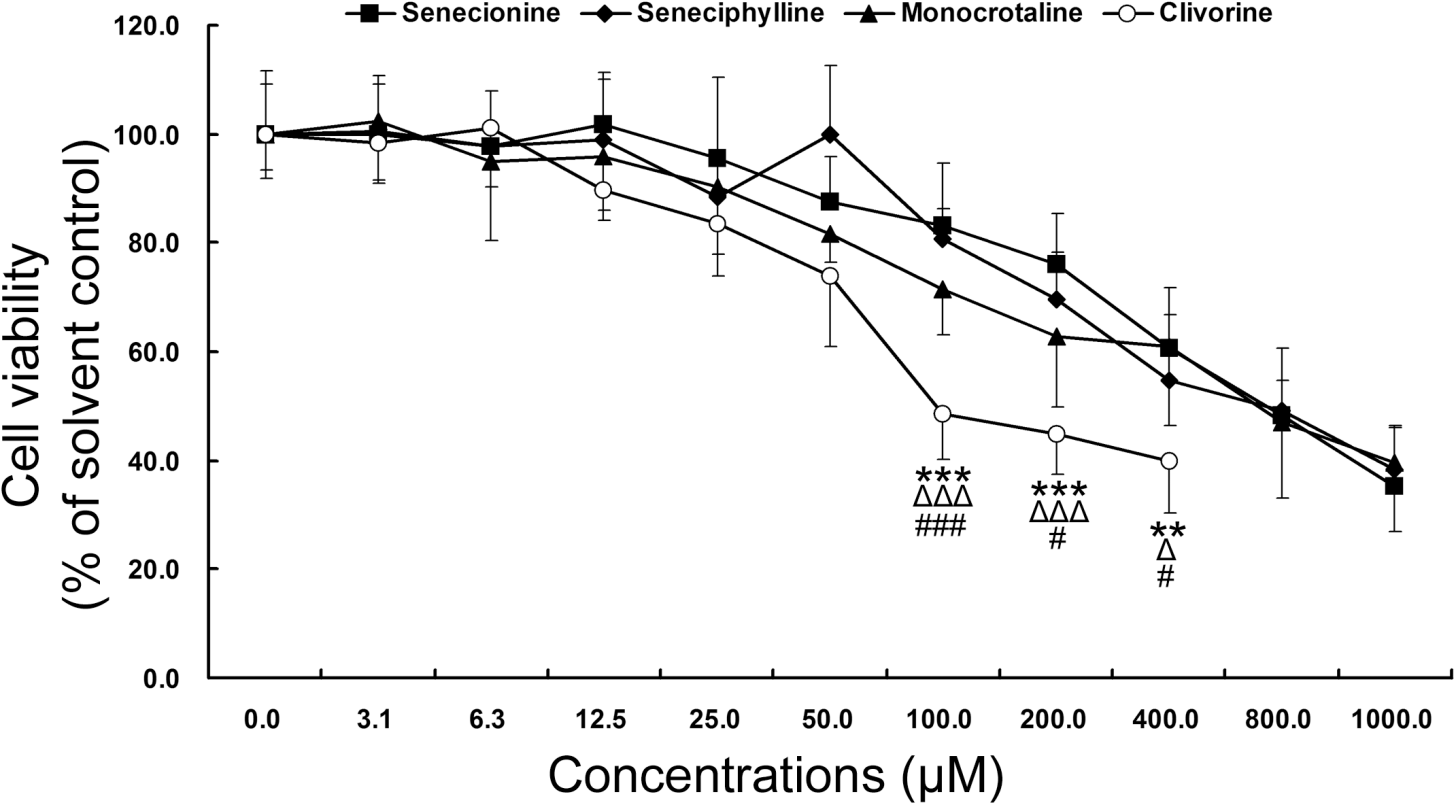
Cytotoxic effects of senecionine, seneciphylline, monocrotaline and clivorine increase with the increase of their concentrations in Huh-7.5 cells as determined by MTT. Cells were incubated with indicated concentrations of senecionine (closed rectangle), seneciphylline (closed diamond), monocrotaline (closed triangle) and clivorine (open circle) for 48 h, respectively. Cell viability was measured by MTT assay as described in Materials and Methods. Values are shown as mean ± SD (n = 3), of which the solvent control are at 0 μM; ^Δ, #^*P* < 0.05, ^**^P < 0.01, ^***, ΔΔΔ, ###^*P* < 0.001, comparing clivorine with senecionine (^*^), seneciphylline (^Δ^), and monocrotaline (^#^), respectively, at the corresponding concentrations.

Senecionine, seneciphylline, monocrotaline and clivorine (Fig 1) are four representatives of hepatotoxic PAs present in many Chinese herbal medicines [3, 9, 11–15]. As shown in Table 1 and Fig 2, all four alkaloids could inhibit the Huh-7.5 cell viability. For each PA, the cell survival rate decreased with the increase of concentration, the efficiency, however, was different. The inhibitory effects of senecionine and seneciphylline in the range of the concentrations from 100 μM to 1000 μM, clivorine from 12.5 μM to 400 μM, monocrotaline from 50 μM to 1000 μM, were substantially higher than the negative control. Comparatively, the otonecine-type PA clivorine showed the highest anti-proliferative potency. At 100 μM for example, its average inhibition rate (51.4%) was significantly higher than 16.7%, 19.3% and 28.4% of other three retronecine-type PAs senecionine, seneciphylline and monocrotaline, respectively. Moreover, the IC_50_ value calculated in average for clivorine was 141.7 μM, which was remarkably lower than those observed with the other three PAs (509.7 μM, 491.9 μM and 413.2 μM). This result was comparable to that reported [4, 20], in which clivorine exhibited the higher cytotoxicity than other types of PAs. Because MTT assay is a common method for assessing the inhibitory effect on cell viability, our result further demonstrated that direct cell death may play an important role in the toxicity of clivorine. It is worth of note that the IC_50_ values obtained in different studies may be largely different from each other, *e*.*g*., the IC_50_ value for clivorine in this study was much higher than 40.8 μM in another study by Ji *et al*. [20]. The discrepancies may be due to differences in cell lines being used or *in vitro* model for cytotoxicity screening [4, 20]. The Huh-7.5 cell line used in the present study was derived from Huh-7 cell line, which has been reported to have no or mutant expression of *bcl-2* and *p53* genes, indicating that this cell line may be susceptible to toxins but insensitive to some cytotoxic agents-induced apoptosis [20, 21]. Because different types of PAs may have different modes of cytotoxic effects [4, 20, 23], the toxic responses and underlying mechanisms of Huh-7.5 cell line to various PAs with different types or necine acid moieties may be different. On the other hand, considering that the PAs with high concentrations may have more direct cytotoxicity [4, 21], it is also interesting to know how the cells respond to PAs at low or nontoxic concentrations. Accordingly, the maximal non-toxic concentrations (IC_0_s) and those with 10% and 20% of inhibition rates (IC_10_s and IC_20_s) were calculated for all four PAs (Table 1). In comparison, clivorine had the lowest IC_0_, IC_10_ and IC_20_ values of 5.4 μM, 13.4 μM and 27.2 μM in average, respectively. The results especially at the concentrations of IC_20_ or above showed remarkable discrepancy between clivorine and all other retronecine-type PAs. Of note, monocrotaline showed comparable cytotoxic potential to that of clivorine at the lower concentrations than IC_20_. The potency order of hepatotoxicity was thus clivorine ≥ monocrotaline ≥ seneciphylline ≥ senecionine.

**Table 1:** Inhibitory effects of different PAs on cell viability in Huh-7.5 cells.

PA	IC_0_ (μM)	IC_10_ (μM)	IC_20_ (μM)	IC_50_ (μM)
Senecionine	22.7±10.0 ^**^ ^##^	57.2±22.1 ^**^ ^##^	136.7±59.3 ^**^ ^#^	509.7±122.6 ^***^
Seneciphylline	21.0±18.3	47.5±28.8 ^*^	113.4±38.1 ^**^ ^#^	491.9±78.0 ^***^
Monocrotaline	5.9±2.6	20.1±5.1	59.9±12.2 ^**^	413.2±80.6 ^***^
Clivorine	5.4±3.6	13.4±8.4	27.2±14.7	141.7±28.3

All values expressed as Mean ± SD (n = 3).

^*^P<0.05

^**^P<0.01 and

^***^P<0.001 comparing with clivorine

^#^*P*<0.05

^##^*P*<0.01 comparing with monocrotaline.

### Senecionine, seneciphylline, monocrotaline and clivorine induce apoptosis of Huh-7.5 cells

**Fig 3:**
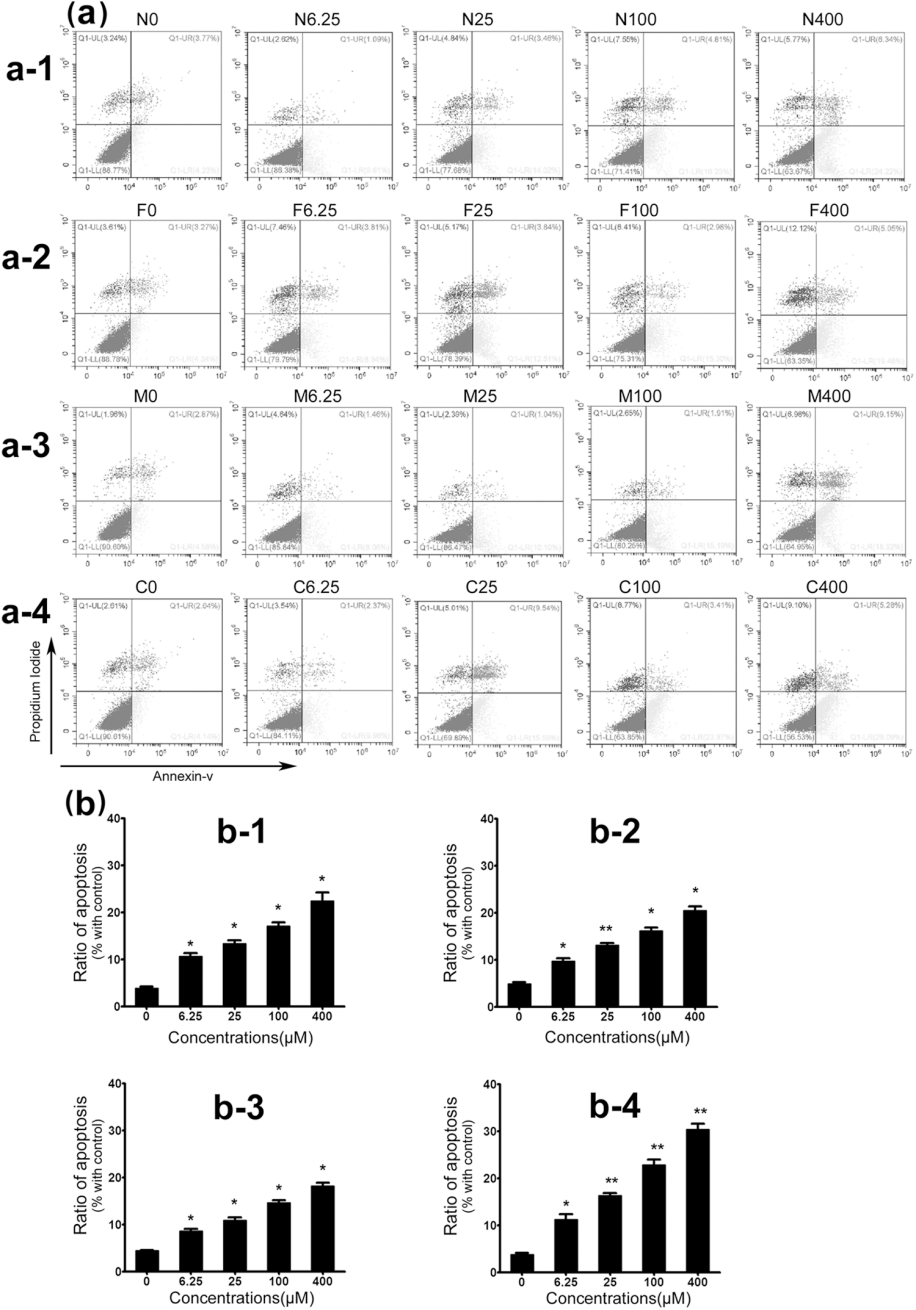
Evaluation of senecionine, seneciphylline, monocrotaline and clivorine-induced apoptosis of Huh-7.5 cells by flow cytometry analysis. Cells were treated by four concentrations of senecionine, seneciphylline, monocrotaline and clivorine for 24 h, respectively. Afterwards, the cells were collected and stained with Annexin V-kFluor488 and PI. The flow cytometry analysis was performed. The percentages of apoptotic cells at both the early and late periods were shown as Q1-LR and Q1-UR, respectively, in the form of scatter plots (**a-1** to **a-4**). The results in the representative of three independent experiments were marked in each diagram and summarized in the histograms (**b-1** to **b-4**). **a-1** and **b-1** show the results from senecionine-treated cells; **a-2** and **b-2**, from seneciphylline-treated cells; **a-3** and **b-3**, from monocrotaline-treated cells; **a-4** and **b-4**, from clivorine-treated cells. N0, F0, M0 and C0, negative controls; N6.25, N25, N100 and N400 represent senecionine-treated cells at the concentrations of 6.25 μM, 25 μM, 100 μM and 400 μM; F6.25, F25, F100 and F400 represent seneciphylline-treated cells at the concentrations of 6.25 μM, 25 μM, 100 μM and 400 μM; M6.25, M25, M100 and M400 represent monocrotaline-treated cells at the concentrations of 6.25 μM, 25 μM, 100 μM and 400 μM; and C6.25, C25, C100, and C400 represent clivorine-treated cells at the concentrations of 6.25 μM, 25 μM, 100 μM and 400 μM, respectively. ^*^P<0.05; ^**^P<0.01 vs. negative control.

As for the mechanism of cytotoxicity for PAs, many studies suggested that the apoptotic cell death may play a vital role in it [13, 20–23]. Senecionine, monocrotaline and clivorine had been found to induce apoptosis in several liver cell lines, to the exclusion of Huh-7.5 cells. In this experiment, the effects of four PAs including senecionine, seneciphylline, monocrotaline and clivorine on the apoptosis of Huh-7.5 cells were further investigated and compared by using flow cytometry. Based on the above analyses, a series of concentrations around or lower than IC_50_s were investigated. As a result, the overall apoptosis ratios or AIs of Huh-7.5 cells increased with increasing concentrations of each PA (Fig 3). All three retronecine-type PAs, senecionine, seneciphylline and monocrotaline showed almost same potency to induce the apoptosis in Huh-7.5 cells. Generally, their AIs against Huh-7.5 cells at all concentrations determined (6.25 μM, 25 μM, 100 μM and 400 μM) were markedly higher than that of the negative control (4.2% in average), but all these values were not very high (around 20% in average at 400 μM) and showed no differences from each other at the same concentrations. This result suggests that the concentration may contribute to their potencies to induce apoptosis.

In comparison, clivorine produced relatively higher apoptosis ratios of Huh-7.5 cells than other three retronecine-type PAs at the corresponding concentrations. The discrepancies become much more obvious at the concentrations higher than 25 μM, in which the AI value at 100 μM (22.8 ± 1.7%) was almost equal to or higher than those of senecionine, seneciphylline and monocrotaline at 400 μM and considerably higher than those of them at the same concentration. This effect may partially be derived from the direct cytotoxicity of clivorine, but for the retronecine-type PAs may mainly from the inhibition of cell proliferation [4]. Except for the concentration, the chemical structure especially necine bases may also be a key determinant for the PA-induced apoptosis. Based on above experiment, clivorine may have higher potency to induce apoptosis in Huh-7.5 cells than other PAs, but which effects tend to be consistent at the low concentrations of 25 μM (16.2 ± 0.9%) and 6.25 μM (11.2 ± 1.7%). Also, monocrotaline did not show apparent high effects comparable to those in MTT assay at the concentrations less than IC_20_. These results suggest that apoptosis may not be mere determinant for the PA-induced cytotoxicity, in which some other mechanism of action may be involved. Combined the results from MTT, the PAs intoxication may depend on their chemical structures and concentrations, which was in good agreement with those reported by previous investigators [4, 21].

### Senecionine, seneciphylline and clivorine enhance the mRNA expression of LC3 in Huh-7.5 cells

**Fig 4:**
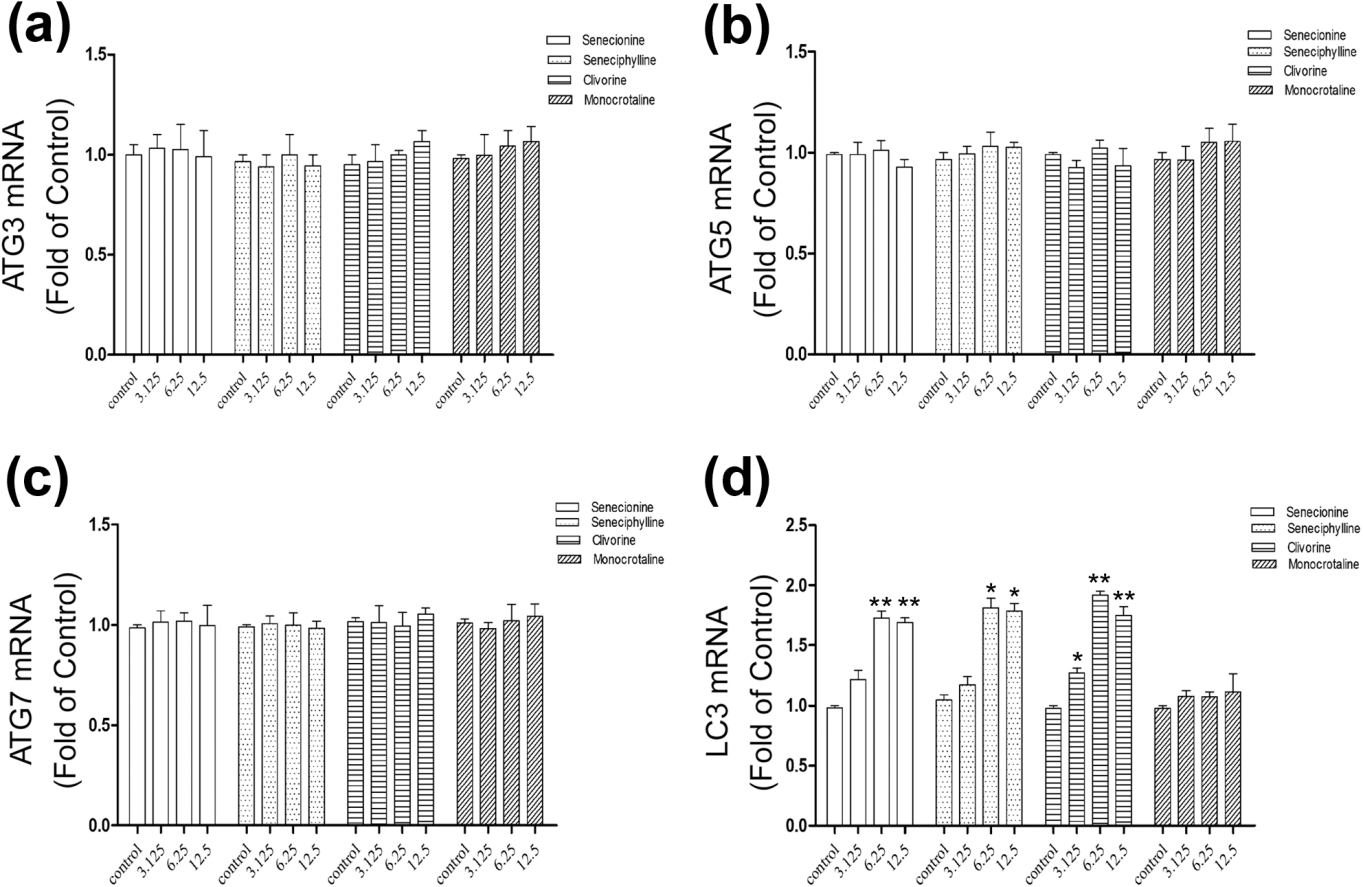
Differential effects of senecionine, seneciphylline, clivorine and monocrotaline on the expression of the autophagy-associated genes. Huh-7.5 cells were cultured in the presence or absence of senecionine, seneciphylline, clivorine and monocrotaline (3.125 μM, 6.25 μM and 12.5 μM) for 24 h. The relative expression levels of four autophagy-associated genes, that is *ATG3* (**a**), *ATG5* (**b**), *ATG7* (**c**) and *LC3* (**d**) in Huh-7.5 cells were determined by RT-PCR with normalization to corresponding GAPDH, and expressed as the fold of control (with DMEM treatment, which was defined as 1). All variables in each experiment were tested in triplicate. ^*^P<0.05; ^**^P<0.01 vs. control.

The PA-induced cytotoxicity in Huh-7.5 cells markedly decreased at low or even nontoxic concentrations, which may not be causally involved by apoptosis or necrosis. In this scenario, some reports suggested that the cell defence but not direct toxic pathway may play a pivotal role [32–34]. Under stressful conditions, autophagy is usually activated to protect the cell from damage [38]. A variety of key autophagy-relevant components have been found to participate in the activation by involving in ubiquitin-like conjugation systems, for instance, Atg3, Atg5, Atg7 and LC3 involved in the LC3 lipidation and ATG12-ATG5 conjugation systems that are essential for the autophagosome formation and maturation [39]. Their transcriptional and translational regulation especially that of LC3, the most widely used marker for monitoring autophagy is preferred to provide correlative data related to autophagy [27, 37–39]. Accordingly, four autophagy-associated genes including *ATG3*, *ATG5*, *ATG7* and *LC3* were examined by RT-PCR to observe if senecionine, seneciphylline, monocrotaline and clivorine could modulate autophagy at the transcriptional level. To avoid the undesirable direct toxic effects, the choice of the concentration was mainly based on the values in the range of IC_0_ and IC_10_ of clivorine for each PA. As shown in Fig 4, the relative mRNA levels of *LC3* as the fold of control were 1.27 ± 0.06, 1.92 ± 0.05 and 1.75 ± 0.10, in the cells treated by clivorine at 3.125 μM, 6.25 μM and 12.5 μM, 1.73 ± 0.08 and 1.69 ± 0.06 by senecionine at 6.25 μM and 12.5 μM, and 1.81 ± 0.11 and 1.79 ± 0.09 by seneciphylline at the same two concentrations as senecionine, respectively, all of which were significantly higher than that of negative control. For the three PAs, the effects at 6.25 μM and 12.5 μM were also remarkably higher than those at the lower concentration of 3.125 μM. The concentration-dependent effects were obvious but slightly dropped at the high concentration of 12.5 μM. For monocrotaline, no unambiguous statistical significance was observed for all concentrations compared with the control. Moreover, there was no substantial change in the mRNA levels of *ATG3*, *ATG5*, and *ATG7* genes between the PAs-treated and un-treated cells. These results indicated that senecionine, seneciphylline, and clivorine could enhance the mRNA expression of *LC3* in Huh-7.5 cells, in which clivorine was more effective than other twos at low concentration close to its IC_0_ (6.25 μM) or nontoxic concentration (3.125 μM) (see Fig 4). The transcriptional regulation mediated by PAs may be selective for either *ATG* genes or PA structures and concentrations in this setting. The up-regulation of the mRNA level of *LC3* has been documented to be involved in the elevated autophagy flux probably by replenishing the LC3 protein destroyed during autophagosome fusion with the lysosome [39, 40].

### Senecionine, seneciphylline and clivorine induce autophagy in Huh-7.5 cells

**Fig 5:**
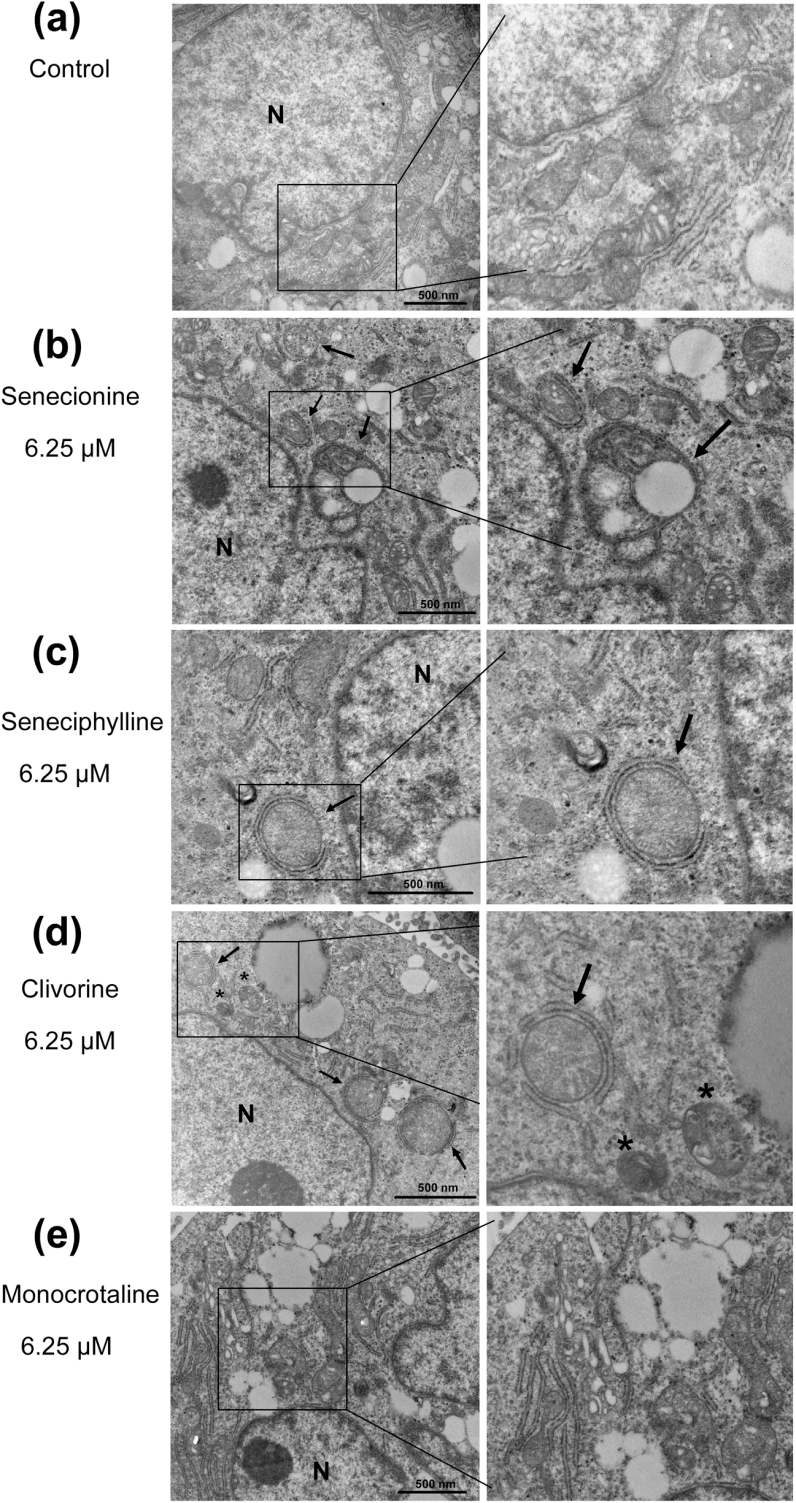
Transmission electron micrographs of autophagosome induced by senecionine, seneciphylline, clivorine and monocrotaline in Huh-7.5 cells. (**a**) The untreated Huh-7.5 cells (Control); (**b**) The senecionine -treated cells (6.25 μM), (**c**) The seneciphylline-treated cells (6.25 μM), (**d**) The clivorine-treated cells (6.25 μM), (**e**) The monocrotaline-treated cells (6.25 μM). Huh-7.5 cells were cultured in the presence of senecionine, seneciphylline, clivorine and monocrotaline for 24 h, respectively. As indicated by black arrows, the autophagic vacuoles with double-membrane structures were observed in cytoplasm. Asterisks indicate mature autophagosomes containing highly degraded contents. N, nucleus. Scale bar, 500 nm.

**Fig 6:**
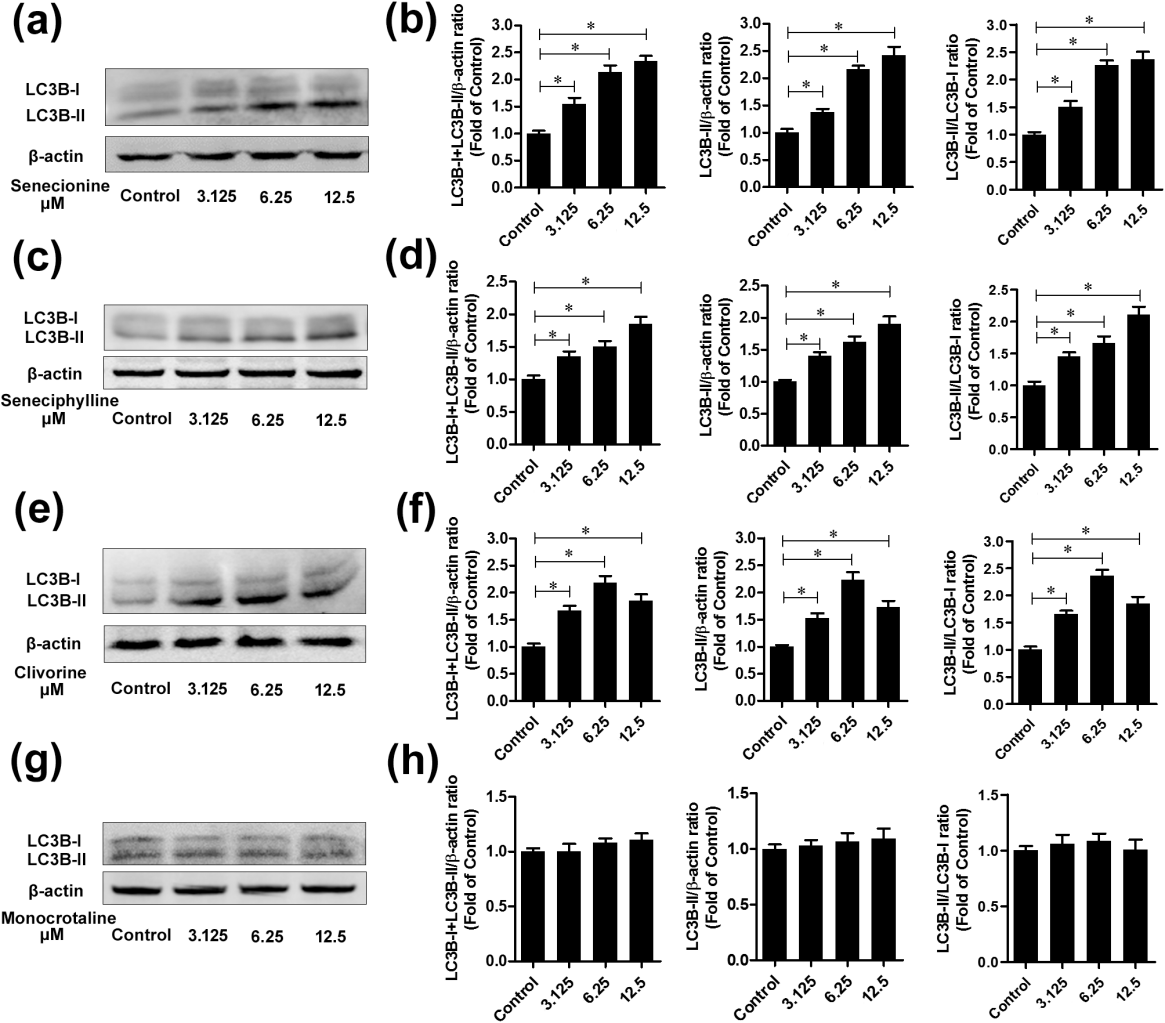
The LC3B expression in Huh-7.5 cells treated by senecionine, seneciphylline, clivorine and monocrotaline. Huh-7.5 Cells were incubated in the presence or absence of senecionine, seneciphylline, clivorine and monocrotaline with various concentrations for 24 h. 3.125 means the cells were treated with PAs at 3.125 μM; similarly, 6.25 with PAs at 6.25 μM and 12.5 with PAs at 12.5 μM. The control cells were treated with solvent only. Total cellular proteins were collected and subjected to western-blot using the antibodies against LC3B-I, LC3B-II and β-actin (internal control). (**a, c, e, g**) Representative western blots of LC3B-I, LC3B-II and β-actin are shown. (**b, d, f, h**) The densitometric intensities of LC3B-I, LC3B-II and β-actin bands were quantified by image J software. The relative LC3B-I+LC3B-II/β-actin, LC3B-II/β-actin and LC3B-II/LC3B-I ratios were calculated and shown as the fold of control (without PAs treatment under different groups, which was defined as 1, respectively). The data shown in Figs 6b, 6d, 6f, and 6h are the mean ± SD of the results of three independent experiments. ^*^P<0.05 vs. control.

Based on the above results, autophagy may mediate the PA-induced toxicities in Huh-7.5 cells. To further clarify this effect, we first examined the formation of autophagosomes under transmission electron microscopy (TEM). As can be seen in Fig 5, the closed double-membrane structures or autophagic vacuoles (with a diameter of 300 to 900 nm) were distinctly observed around the nucleus in cells treated by 6.25 μM of senecionine, seneciphylline and clivorine, in comparison with mainly rough endoplasmic reticulum (ER) and mitochondria in the similar region in the untreated cells; also in this concentration clivorine treatment generated some mature autophagosomes containing sequestered materials. In the monocrotaline-treated cells, however, the similar morphological feature of autophagic vacuole accumulation was not observed (Fig 5E). Then we measured the expression of the autophagy marker, autophagosomal membrane protein LC3B, to assess the autophagic activation. The total expression of this protein and the ratio of LC3B-II and LC3B-I, may be elevated owing to increased autophagy induction [37, 39]. Comparatively, LC3B-II is more specific than LC3B-I for the assessment of autophagosomes or autophagic flux, which correlation has been demonstrated in many studies of natural compounds involving cytoprotective autophagy [27]. In this study, the protein levels of LC3B-I and LC3B-II were determined by western-blot analysis after exposure to each PA, of which three concentrations (3.125 μM, 6.25 μM and 12.5 μM) were selected between the IC_0_ and IC_10_ values of clivorine to exclude high toxic disturbances on Huh-7.5 cells. Except for clivorine, senecionine and seneciphylline both largely increased the transcriptional expression of *LC3* at high concentrations of 6.25 μM and 12.5 μM but monocrotaline had no effect at any concentration (Fig 4). As a comparison, it was also interesting to know how they affect the protein expression of LC3B especially LC3B-II in Huh-7.5 cells. As can be seen from Fig 6A–6F, treatment with senecionine, seneciphylline and clivorine all resulted in remarkable increases of the relative protein levels of either total LC3B or LC3B-II at the tested concentrations compared with the negative control. Correspondingly, the relative LC3B-II/LC3B-I ratios were also up-regulated remarkably at all treatments. Unlike these results, however, monocrotaline had no any effect (Fig 6G and 6H). This evidence suggests that clivorine, senecionine and seneciphylline but not monocrotaline could induce autophagy in Huh-7.5 cells. It was noteworthy that the induction by senecionine and seneciphylline were in a concentration-dependent manner but clivorine showed a different trend. From Fig 6E and 6F, the accumulation of either total LC3B or LC3B-II reached the highest level at 6.25 μM but declined at 12.5 μM. Similarly, the relative LC3B-II/LC3B-I ratios significantly increased and also peaked at 6.25 μM in the clivorine-treated cells. This distinct effect by clivorine may be related to its higher toxic potency at the concentrations used in the assay than other PAs (Table 1).

**Fig 7:**
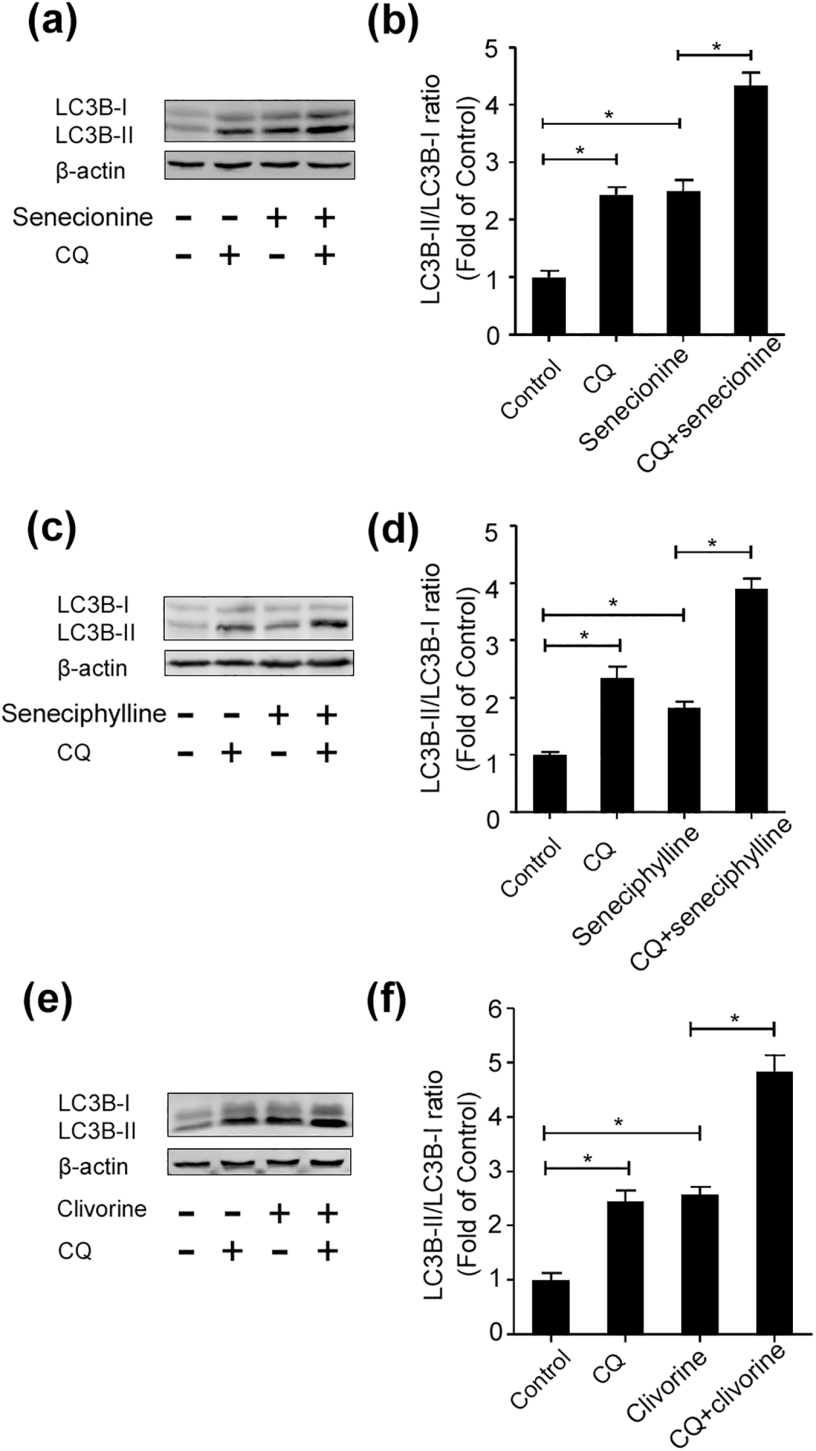
Senecionine, seneciphylline and clivorine activate autophagic flux in Huh-7.5 cells. (**a, c, e**) A representative western blot image showing LC3B protein expression in different groups. Senecionine (6.25 μM), seneciphylline (6.25 μM), clivorine (6.25 μM) treated or control Huh-7.5 cells were cultured in the presence or absence of CQ (5 μM, 2 h pre-incubation) for 24 h. The cellular proteins were extracted for Western-blot analyses. (**b, d, f**) Quantitative assessment of LC3B-I and LC3B-II protein expression in different groups. The densitometric intensities of LC3B-I, LC3B-II and β-actin bands were quantified by Image J software. The relative LC3B-II/LC3B-I ratios were calculated and shown as the fold of control (without PA treatment under different conditions, which was defined as 1, respectively). The data shown in Fig 7b, 7d, and 7f are the mean ± SD of the results of three independent experiments. The *P* value was calculated by Student’s *t*-test (^*^P < 0.05).

**Fig 8:**
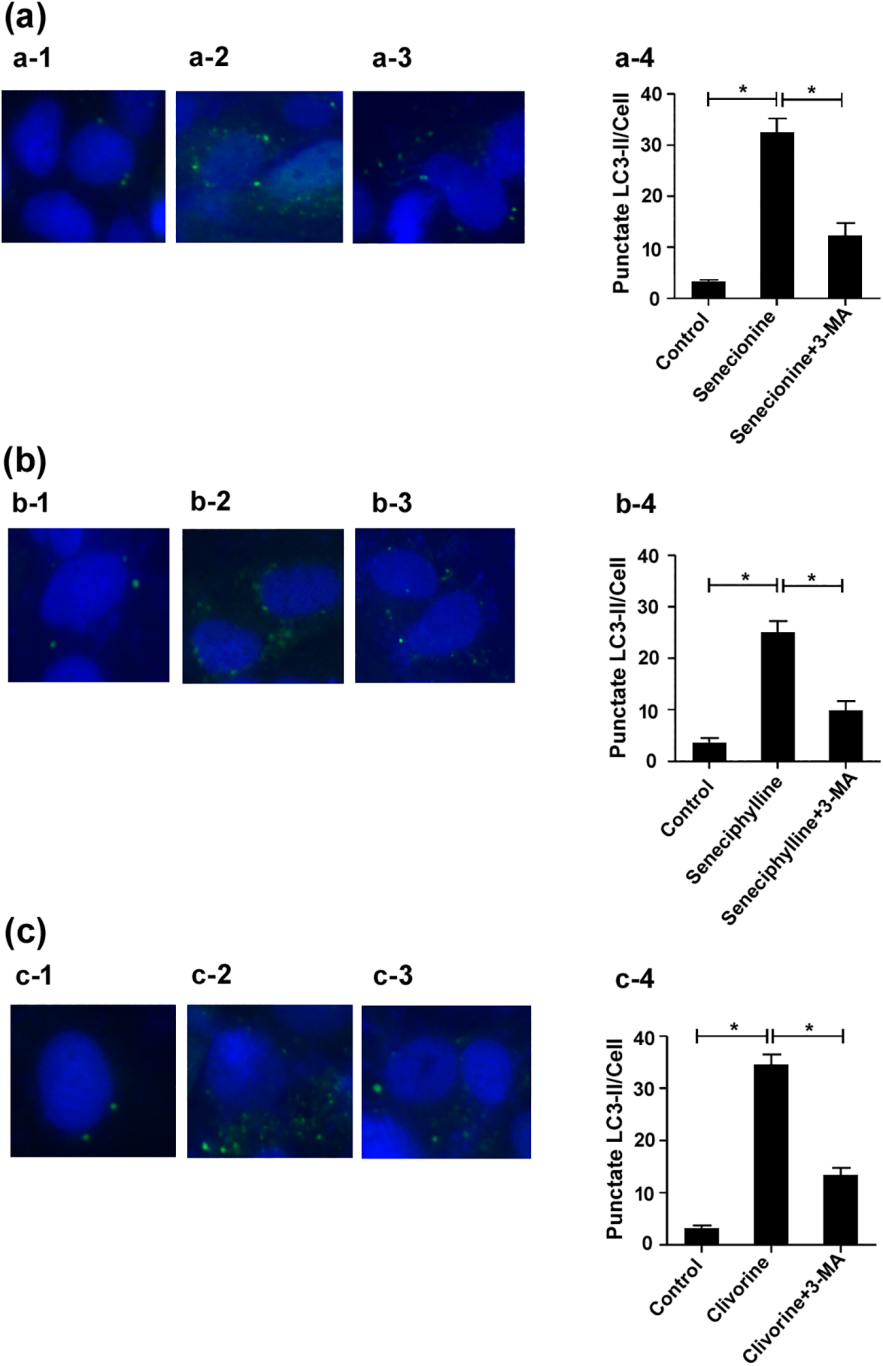
The effects of senecionine, seneciphylline and clivorine on GFP-LC3 puncta in Huh-7.5 cells. Huh-7.5 cells expressing the GFP-LC3 protein were established by using lentivirus. The cells were being observed under a fluorescent microscope, **a-1,** the cells were untreated with PA; **a-2,** the cells were treated with senecionine (6.25 μM) alone for 24 h; **a-3,** the cells were treated with senecionine (6.25 μM) for 24 h after 2 h pre-incubation with the autophagy inhibitor 3-methyladenine (3-MA, 5 mM). **b-1**, the cells were untreated with PA; **b-2**, the cells were treated with seneciphylline (6.25 μM) alone for 24 h; **b-3**, the cells were treated with seneciphylline (6.25 μM) for 24 h after 2 h pre-incubation with 3-MA (5 mM). **c-1**, the cells were untreated with PA; **c-2**, the cells were treated with clivorine (6.25 μM) alone for 24 h; **c-3**, the cells were treated with clivorine (6.25 μM) for 24 h after 2 h pre-incubation with 3-MA (5 mM). **a-4**, **b-4**, **c-4**, GFP-LC3 puncta per transfected cell were determined (30 cells were counted per experiment). Data shown are mean ± SD of the results of three independent experiments. The *P* value was calculated by Student’s *t*-test (^*^P < 0.05).

Since the change of LC3B-II level could be caused by either autophagosome formation or degradation in lysosomes, we further investigated senecionine, seneciphylline, and clivorine-induced autophagic flux in the presence and absence of CQ (5 μM), a lysosomal inhibitor that may block the fusion of autophagosomes with lysosomes [39]. The Western blot analysis showed that both the PAs and CQ remarkably induced conversion of LC3B-I to LC3B-II in Huh-7.5 cells, respectively, while their co-treatments with CQ further enhanced the conversion evoked by CQ alone (Fig 7). This suggests that the senecionine, seneciphylline, and clivorine-induced increase in LC3B-II levels may be ascribed to the increased autophagosome formation. Furthermore, we assessed the formation of punctate structures (puncta) with GFP-LC3 reporter, a marker of autophagosomes formation that represents the accumulation of LC3B-II on autophagic vacuoles [39, 41]. As a result, a significant increase of cells containing GFP-LC3 puncta was observed in Huh-7.5 cells treated with senecionine, seneciphylline, and clivorine for 24 h, respectively, and the non-specific sequestration inhibitor 3-MA (5 mM) partially blocked the increase of GFP-LC3 puncta per transfected cell induced by them (*P* < 0.05) (Fig 8). Collectively, these data indicated that senecionine, seneciphylline and clivorine induced autophagy in Huh-7.5 cells especially at the concentrations close to the IC_0_ value of clivorine. Moreover, these results suggest that the structural differences in either necine base or necic acid groups of these PAs (Fig 1) may contribute to their differential effects on autophagy in Huh-7.5 cells.

### Autophagy protects Huh-7.5 cells from PAs-induced apoptotic cell death

**Fig 9:**
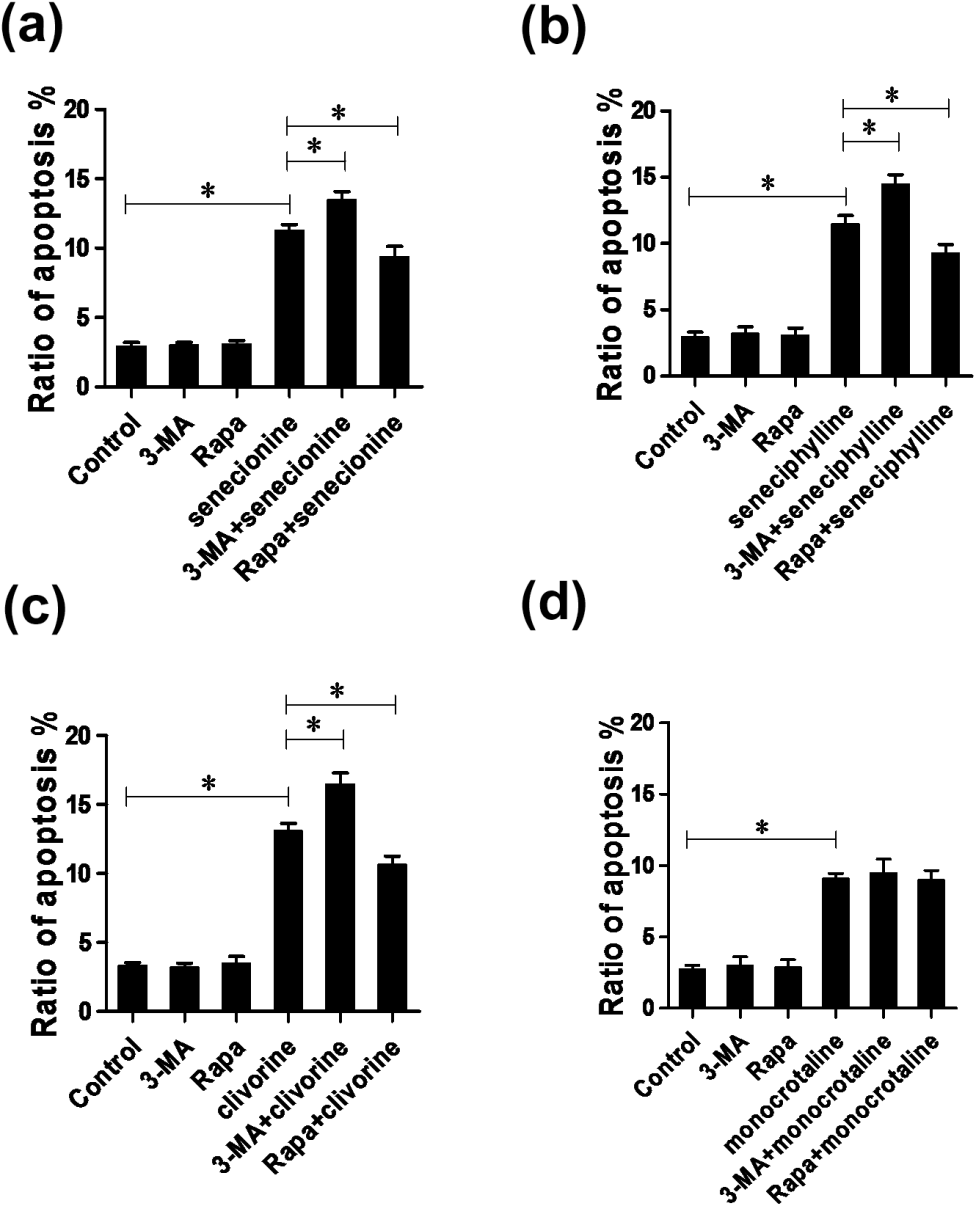
Effects of autophagy inhibitor or activator on PAs-induced cell apoptosis. (**a**) Senecionine (12.5 μM), (**b**) seneciphylline (12.5 μM), (**c**) clivorine (12.5 μM) and (**d**) monocrotaline (12.5 μM) treated or untreated Huh-7.5 cells were pre-cultured in the presence of autophagy inhibitor 3-MA (5 mM)) or activator Rapa (100 nM) for 2 h, and then cell apoptosis were subjected to flow cytometry analyses. The data shown in 9a, 9b, 9c and 9d are the mean ± SD of the results of three independent experiments. The *P* value was calculated by Student’s *t*-test (^*^P < 0.05).

Apoptosis and autophagy are two important and interconnected processes and play important roles in the promotion or inhibition of cell survival in response to many natural products or chemotherapeutic drugs [27, 33, 40–42]. From the above studies, both apoptosis and autophagy were involved in the PA-induced cellular responses. In order to clarify the relationship between apoptosis and autophagy, the role of autophagy in the PA-treated cell survival was further investigated. After pre-treatment with the autophagy inhibitor 3-MA (5 mM) or autophagy activator Rapa (100 nM), Huh-7.5 cells were incubated with senecionine, seneciphylline, clivorine and monocrotaline for 24 h, respectively. Afterwards, the cell apoptosis was examined. As shown in Fig 9, the Huh-7.5 cell apoptosis induced by senecionine, seneciphylline, clivorine and monocrotaline was all obvious at the concentration of 12.5 μM. The 3-MA treatment (for autophagy suppression) significantly enhanced senecionine, seneciphylline and clivorine-induced cell apoptosis compared with each PA alone, whereas the Rapa treatment (for enhancement of autophagy) markedly weakened these PAs-induced apoptosis. In addition, we did not observe that either 3-MA treatment or Rapa treatment altered monocrotaline-induced apoptosis. These results indicated that autophagy may have a protective role to prevent the hepatic cells from PAs-induced apoptosis.

## Discussion

Autophagy as a highly conserved cellular homeostatic process was found to be regulated by many natural products to make either protective or cytotoxic effects across different types of mammalian cells [27]. It may be difficult to exactly predict which effects will be dominant because the autophagic process may not exist alone, but interconnected with many other cellular processes. For example, autophagy and apoptosis can interact on each other in an inhibitory way through some common proteins and signalling pathways; autophagy usually exhibits a dual role in cell fate determination [39–42]. Previous studies have shown that some PAs can induce apoptosis in isolated mouse hepatocytes [20], human liver L-02 cells [20, 24] and human hepatoma cells HepG2 [4] and Huh-7 [21]. In this study, we tested and proved the differential toxic effects of PAs with different types or structures and concentrations on Huh-7.5 cells. In combination with the previous studies, we propose the following hypotheses: (1) PAs induce cytotoxicity by involving in apoptosis and/or necrosis pathways, which may be linked to autophagy; (2) The induction of autophagy may be concentration and structure-dependent; (3) The initial autophagy may have a positive role in the cell survival. Using four PAs with two types, we demonstrated firstly that two retronecine-type PAs, senecionine and seneciphylline, and a representative otonecine-type PA clivorine, did induce autophagy in Huh-7.5 cells at the concentrations close to the IC_0_ and IC_10_ values of clivorine via RT-PCR, western-blot, fluorescence microscopy and transmission electron microscopy analyses. This effect was accompanied by an up-regulation of the LC3 pathway and accumulation of autophagosomes (Figs 4–8). On the one hand, it may be concentration-dependent because in many other studies, the high concentrations were usually used to explore the toxic mechanism of PAs, such as 50 μM and 100 μM for clivorine [7, 20] and 500 μM for monocrotaline [8], in which no autophagy but apoptosis or necrosis (depending on treatment time) was definitely observed. According to our results, the clivorine-induced autophagy occurred at its nontoxic concentration of 3.125 μM, developed or peaked at 6.25 μM (a concentration around its IC_0_ value), and decreased at 12.5 μM (around IC_10_); on the contrary, apoptosis become prominent at 6.25 μM and increased with the increase of concentration (Figs 3 and 6). The concentration around IC_0_ may be a turning point to switch autophagy to apoptotic cell death. This effect was distinct for clivorine amongst the tested PAs because senecionine and seneciphylline increased the LC3B protein expression in a concentration-dependent manner whereas monocrotaline had no effect at all concentrations used in the assay (Fig 6). Meanwhile, at these concentrations clivorine displayed the higher toxic potency than other PAs (Table 1, Fig 2). All the evidence suggests that concentration may have a crucial role in the PA-induced autophagy especially by clivorine. The long-recognised mechanism of the PA-induced cytotoxicity involving metabolic activation by CYP450s may play a key role in this effect [17–19]. According to the existing findings about CYP450 activities in Huh-7 cells [43] and further study on the in vitro metabolism of clivorine in pooled human liver microsomes (S1 File), however, we found that the metabolic activation by CYP450s in our Huh-7.5 cell culture system may be too weak to generate a certain level of reactive “pyrrolic” metabolites to make the cellular responses we have observed. Nevertheless, how much exactly the pyrrolic metabolites can be formed at the levels of exposure used in our Huh-7.5 cell culture system and to what extent the metabolic activation and associated CYP450s involving formation of these metabolites is contributing to the autophagy or other cytotoxic effects of PAs warrant further investigation.

On the other hand, the structural factor may also be critical for the PA-induced autophagy. In this study, we used four PAs including three retronecine-type PAs senecionine, seneciphylline and monocrotaline and one otonecine-type PA clivorine, all with different necic acids (Fig 1). Even in the same type, senecionine and seneciphylline are 12-membered macrocyclic diesters while monocrotaline is an 11-membered one. The structural difference may lead to different physicochemical property (*e*.*g*., lipophilicity) and/or steric effects, which eventually affect the hepatotoxic potential of PAs [4]. Our study demonstrated that monocrotaline induced apoptosis alone, while clivorine, senecionine and seneciphylline could induce both apoptosis and autophagy. Clivorine can more readily inhibit cell viability, and induce apoptosis than other PAs. Clivorine treatment at its IC_0_ level of exposure (6.25 μM) showed the highest autophagic response but flattened ratio of apoptosis, which was different from other retronecine-type PAs (Figs 3 and 6). This result suggests a distinct mechanism for the cell responses to clivorine. Monocrotaline showed the highest inhibitory effects against Huh-7.5 cells among the three retronecine-type PAs but had less apoptotic potency at the concentrations tested, which may rest with activation of other cell death processes such as necrosis [8, 22]. The deficiency of autophagy induction by monocrotaline may also partially contribute to its high cytotoxicity at the low levels of exposure. Overall, autophagy is involved in the molecular machinery of PA-induced cytotoxicity in Huh-7.5 cells, which activation/regulation may depend on concentration and structure of PAs.

In addition, the clivorine-induced autophagy as mentioned above may be protective because clivorine at the concentration level close to IC_0_ had much less anti-proliferative effects (Fig 2) and apoptosis-inducing potencies on Huh-7.5 cells than those at the high concentrations (Fig 3). More importantly, the treatment by autophagic inhibitor or activator could significantly enhance or weaken clivorine-induced apoptosis at 12.5 μM, respectively (Fig 9). Since this concentration was close to the IC_10_ for clivorine (Table 1), showing some degree of direct cytotoxicity and apoptosis (Figs 2 and 3), the regulation of autophagy may have important implication for the cell death/defence responses to this PA (Fig 9). As for the other tested PAs, senecionine and seneciphylline also showed the similar evidence at the same concentration, which is far below the IC_0_s of two PAs (Table 1). In the context of stress conditions, autophagy may be an adaptive response earlier than apoptotic cell death [27, 33]. Therefore, the autophagic activation rather than apoptosis or necrosis is likely to serve as a pro-survival mechanism in the initial stage of PA-induced cytotoxicity. In combination with the toxic effects from the PAs, the multiple cell survival and death pathways linking autophagy, apoptosis and necrosis may be intermingled [33, 40].

In summary, the present study investigated the effects of three retronecine-type PAs (senecionine, seneciphylline and monocrotaline) and one otonecine-type PA (clivorine) on the proliferation of Huh-7.5 cells and further explored potential cellular and molecular bases on it. Our results indicated that PAs with different types or structures and concentrations may exert different toxic disturbances on Huh-7.5 cells. Apoptosis may be the common cause of cell death shared by them. In comparison, clivorine is the most toxic PA due to its highest direct cytotoxicity and apoptosis-inducing potency. Most notably, clivorine can induce autophagy in Huh-7.5 cells at the concentrations close to its IC_0_ and IC_10_ values by up-regulating the expression of autophagy markers LC3B in both transcriptional and protein levels as well as autophagosome accumulation. Another two PAs including senecionine and seneciphylline also induced the protective autophagy at the low level of exposure. The autophagy pathway may serve as a protective mechanism in the early stage of PA intoxication. The differential induction of apoptosis and autophagy by clivorine probably depending on the concentration may play an essential role in its cytotoxic potency. This work provides a new mechanistic insight into the cell death/survival modality mediated by PAs or PA-containing products. Also, our findings suggest that the intrinsic cellular signalling pathways of the PA-induced autophagy and interactions with other important cellular processes deserve further exploration for a comprehensive understanding of their toxic implications.

## Supporting information

S1 File In vitro microsomal metabolism of clivorine by human liver microsomes. In vitro microsomal metabolism of clivorine was investigated using pooled human liver microsomes (*InVitro*CYP^TM^ H-class 25-donor mixed gender) in the presence or absence of NADPH. Microsomal incubations were conducted in a total volume of 200 μl containing human liver microsomes (1 mg protein/mL), NADPH (1 mM), GSH (2 mM) and clivorine (0.25 mM), all in phosphate buffered saline at pH 7.4. The incubation mixture was treated with an equal volume of acetonitrile and centrifuged, and then the supernatant was subjected to HPLC analysis. Representative analytical results are shown in the file, and the experimental details about materials and methods, results and discussion are also presented.
(DOC)
