# Supplementary material for: Differential induction of apoptosis and autophagy by pyrrolizidine alkaloid clivorine in human hepatoma Huh-7.5 cells and its toxic implication
Source: PLoS One. 2017 Jun 26;12(6):e0179379. doi: 10.1371/journal.pone.0179379 (PMC5484491; doi:10.1371/journal.pone.0179379)
Supplement: S1 File — In vitro microsomal metabolism of clivorine was investigated using pooled human liver microsomes (InVitroCYPTM H-class 25-donor mixed gender) in the presence or absence of NADPH. Microsomal incubations were conducted in a total volume of 200 μl containing human liver microsomes (1 mg protein/mL), NADPH (1 mM), GSH (2 mM) and clivorine (0.25 mM), all in phosphate buffered saline at pH 7.4. The incubation mixture was treated with an equal volume of acetonitrile and centrifuged, and then the supernatant was subjected to HPLC analysis. Representative analytical results are shown in the file, and the experimental details about materials and methods, results and discussion are also presented. (DOC) [file pone.0179379.s001.doc]

**Supporting Information for**

**Differential Induction of Apoptosis and Autophagy by Pyrrolizidine Alkaloid Clivorine in Human Hepatoma Huh-7.5 Cells and its Toxic Implication**

Wenju Liu^1#^, Xu Li^2#^, Bo Zhou^2#^, Shoucai Fang^2^, Wenzhe Ho^3^, Hui Chen^4^, Hao Liang^2^, Li Ye^2*^, Jun Tang^1*^

^1^ Key Laboratory of Combinatorial Biosynthesis and Drug Discovery (Wuhan University), Ministry of Education, and Wuhan University School of Pharmaceutical Sciences, Wuhan 430071, P. R. China

^2^ Guangxi Key Laboratory of AIDS Prevention and Treatment & Guangxi Collaborative Innovation Centre for Biomedicine, School of Public Health, Guangxi Medical University, Nanning 530021, Guangxi, P. R. China

^3^ Department of Pathology and Laboratory Medicine, Temple University School of Medicine, Philadelphia, PA 19140, United States of America

^4^ Geriatrics Digestion Department of Internal Medicine, The First Affiliated Hospital of Guangxi Medical University, Nanning 530021, Guangxi, P. R. China

^*^Corresponding authors: [tangj0205@whu.edu.cn](mailto:tangj0205@whu.edu.cn) (JT); [yeli@gxmu.edu.cn](mailto:yeli@gxmu.edu.cn) (LY).

^#^These authors contributed equally to this work.

**S1 In vitro microsomal metabolism of clivorine by human liver microsomes.**

**Materials and Methods**

Clivorine was isolated from *Ligularia hodgsonii* Hook and identified by NMR and MS with above 99% purity, as previously described [1]. (±)-6, 7-dihydro-7-hydroxy-1-hydroxymethyl -5H-pyrrolizine (DHP), 7-GSH-DHP, and 7, 9-diGSH-DHP were chemically prepared using monocrotaline by an adaptation of the published methods [2, 3]. Pooled primary human liver microsomes (Lot #LEV) were purchased from Celsis In Vitro Technologies (Baltimore, MD, USA; catalogue number X008064; H-class 25-donor mixed gender). The microsomes were pre-incubated with clivorine at 37 °C for 5 min in the absence of NADPH (Sigma product, 93-100%). The reaction was initiated by addition of NADPH and then incubated under the same condition. The final incubation concentrations were 0.25 mM clivorine, 1 mM NADPH, 2 mM glutathione (GSH) and 1 mg protein/mL liver microsomes in phosphate buffered saline (PBS) at pH 7.4. Control incubation without NADPH or microsomes was also conducted. One aliquot (100 μL) of the incubation mixture was withdrawn at 1 h and combined immediately with 100 μL of acetonitrile. The resultant mixture was centrifuged at 20, 000 × *g* and 4 °C for 30 min. The supernatant (5 μL) were filtered and subjected to HPLC (Waters 600/996 systems) for qualitative and quantitative analyses. An established HPLC method in our lab [4] with minor modifications was used for the data collection. Briefly, the supernatants were loaded onto a Cosmosil 5C18-MS-II column (Nacalai Tesque Inc., Kyoto, Japan, 4.6 mm × 150 mm, 5 μm) with a 5 mM ammonium formate water solution containing 0.05% formic acid/acetonitrile binary mobile phase system, gradient at 1 mL/min and monitored at 230 nm. The gradient profile was as follows: 0~5 min, 98% A; 5~10 min, 98% A~95% A; 10~22 min, 95% A~80% A; 22~35 min, 80% A~70% A; 35~40 min, 70% A. All experiments were performed in triplicate.

Standard curves were generated by plotting the concentrations of standard compounds (clivorine, DHP, 7-GSH-DHP, and 7, 9-diGSH-DHP) in matrix (deactivated microsomes) against peak area. The calibration curves were linear over the concentration range of 1.2~153.1 μM for clivorine, 1.28~40.8 μM for DHP, 17.8~569.5 μM for 7-GSH-DHP and 5.88~188.1 μM for 7, 9-diGSH-DHP, with the correlation coefficients (*R*^2^) for all analyses above 0.999.

The metabolic efficiency was calculated by referring to the formula given by Ruan et al. [5] with a minor modification, that is, metabolic efficiency (%) = (C_0_﹣C_1h_)/C_0_ × 100%, where C_0_ and C_1h_ were the average concentrations of the intact clivorine determined in the incubation mixture without and with NADPH for 1 h, respectively.

**Results and Discussion**

As shown in the S1 Fig, (A) showed a representative HPLC profile of clivorine and three prepared putative metabolites (DHP, 7-GSH-DHP, and 7, 9-diGSH-DHP), which resolution was good for each other (*R*_s_＞1.5); (B) showed a HPLC profile of control incubation without NADPH, in which no any metabolite was detected, and the content of clivorine was determined to be 128.79 ± 7.79 μM (n=3); (C) showed the full incubation with NADPH, the CYP450-mediated metabolism occurred as expected, in which clivorine, DHP and its GSH adduct 7-GSH-DHP were detected and determined to be 102.31 ± 11.31 μM, 1.36 ± 0.08 μM and 4.36 ± 0.25 μM, respectively (n=3). Under this condition, however, almost no 7, 9-diGSH-DHP was detected.

The metabolic clearance of clivorine characterised by the formation of DHP and its conjugate 7-GSH-DHP was calculated to be less than 5% of total administered substrate, that is, (C_DHP_ + C_7-GSH-DHP_)/C_0_× 100%=(1.36 + 4.36)/128.79 × 100%=4.44%, where C_DHP_, C_7-GSH-DHP_ and C_0_ were the concentrations of DHP, 7-GSH-DHP in the full incubation and that of clivorine in the control incubation, respectively. Moreover, the metabolic efficiency was around 21%, that is, metabolic efficiency (%) = (128.79-102.31)/128.79 × 100% = 20.6%.

According to the lot characterization results by Celsis, the pooled human liver microsomes were qualified to have considerable CYP450 activities with the CYP3A4 activity of 661 pmol/min/mg or 264.4 pmol/min/10^6^ hepatocytes at *K*_m_ concentration (calculated as 1-hydroxymidazolam production rate; 1 g of liver tissue is equivalent to 40 mg microsomal proteins [6] or 10^8^ hepatocytes [7]). From the study by Choi et al. [8], the intrinsic clearance of midazolam in differentiated (DMSO-treated) huh7 were found to be about 0.00059 μl/min/10^6^ hepatocytes, in which the metabolic activities by CYP3A4 were 0.87 pmol/min/10^6^ hepatocytes (calculated as 1-hydroxymidazolam production rate). It can be deduced that the metabolic activation rate for clivorine in growing huh-7.5 cells (no DMSO-treated) should be much less than 0.87/264.4=0.329% of that in primary human hepatocytes. Coupled with our present study, the whole production of all potential “pyrrolic” metabolites (including DHP, GSH trapped and protein-bound pyrroles) at the concentration of 3.125 μM may not be higher than 20.6%×0.329%×3.125×1000 = 2.12 nM. Similarly, at the medium concentration (6.25 μM), the whole production of pyrrolic metabolites may not reach 4.24 nM, and at the high concentration (12.5 μM) may not reach 8.48 nM. Considering that the LOQs were found at 1 ng/mL for probe drugs or maybe 6.54 nM for DHP by Choi et al. [8], it may be difficult to measure these pyrrolic metabolites and/or determine the exact level of each one formed in the huh-7.5 cells at the three concentrations of clivorine exposure by using conventional LC/MS/MS instruments. These results suggest that the “pyrrolic” DHP metabolites may be formed in our huh-7.5 cell culture system but in an amount too few to evoke the cellular responses we observed.

**References**

1. Cheng M, Tang J, Gao Q, Lin G. Analysis on clivorine from alkaloid in aqueous extract of Ligularia hodgsonii and its hepatotoxicity in rats. Chin Tradit Herbal Drugs. 2011; 42(12): 2507–2511.
2. Mattocks AR, Jukes R, Brown J. Simple procedures for preparing putative toxic metabolites of pyrrolizidine alkaloids. Toxicon. 1989; 27:561–567.
3. Chen MX, Li L, Zhong DF, Shen SJ, Zheng J, Chen XY. 9-Glutathionyl-6,7-dihydro-1-hydroxymethyl -5H-pyrrolizine is the major pyrrolic glutathione conjugate of retronecine-type pyrrolizidine alkaloids in liver microsomes and in rats. Chem Res Toxicol. 2016; 29: 180−189.
4. Tang J, Cheng M, Hattori M. Pyrrolizidine alkaloid profile in a traditional Chinese herbal medicine Chuan Zi Wan (Ligulariae Radix et Rhizoma) by liquid chromatography/electrospray ionization ion trap mass spectrometry. Anal Methods. 2012; 4: 2797-2808.
5. Ruan J, Yang M, Fu P, Ye Y, Lin G. Metabolic activation of pyrrolizidine alkaloids: insights into the structural and enzymatic basis. Chem Res Toxicol. 2014; 27(6): 1030-1039.
6. Hakooz N, Ito K, Rawden H, Gill H, Lemmers L, Boobis AR, et al. Determination of a human hepatic microsomal scaling factor for predicting in vivo drug clearance. Pharm Res. 2006; 23: 533–539.
7. Wilson ZE, Rostami-Hodjegan A, Burn JL, Tooley A, Boyle J, Ellis SW, et al. Inter-individual variability in levels of human microsomal protein and hepatocellularity per gram of liver. Br J Clin Pharmacol 2003; 56: 433–440.
8. Choi S, Corcoran PBS Jr, Uprichard S, Jeong H. Characterization of increased drug metabolism activity in dimethyl sulfoxide (DMSO)-treated huh7 hepatoma cells. Xenobiotica. 2009; 39(3): 205–217.


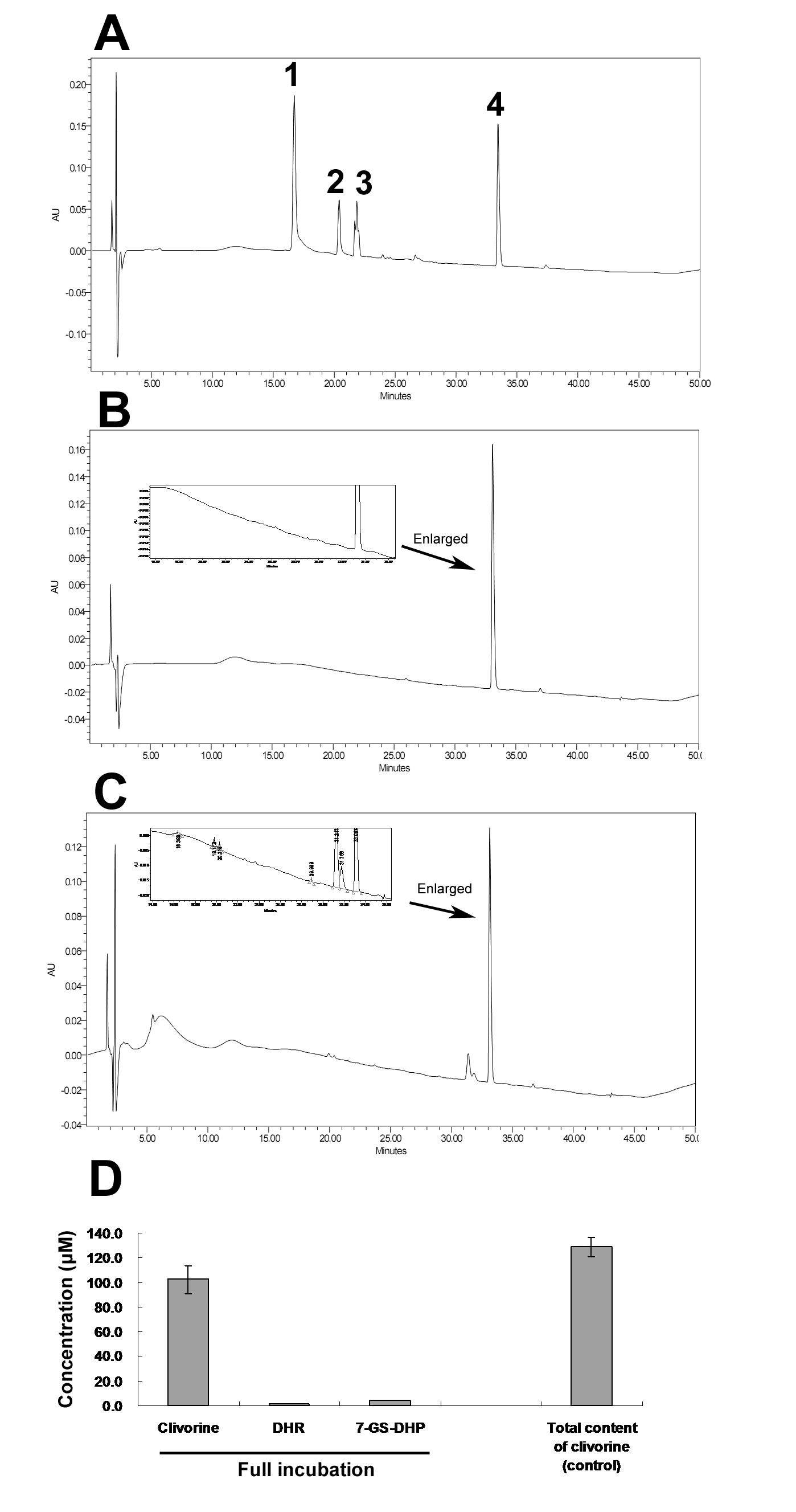


**S1 Fig. In vitro microsomal metabolism of clivorine by human liver microsomes.**

A. The mixed standards with matrix (deactivated microsomes), 1. DHP; 2. 7-GSH-DHP; 3. 7, 9-diGSH-DHP; 4. clivorine; B. Control incubation with matrix (without NADPH) and clivorine (0.25 mM); C. Full incubation for an hour, with pooled human liver microsomes (1 mg/mL, Lot #LEV, from Celsis In Vitro Technologies, Baltimore, MD, USA; catalogue number X008064; H-class 25-donor mixed gender), NADPH (1mM), GSH (2 mM), and clivorine (0.25 mM); D. The data analysis for incubation experiments (n=3). An established HPLC method in our lab with minor modifications was used for the data collection. The details were given in the Materials and Methods.
